# Variation in information needs of patients with interstitial lung disease and their family caregivers according to long-term oxygen therapy: a descriptive study

**DOI:** 10.1186/s12890-023-02795-9

**Published:** 2023-12-05

**Authors:** Ryuhei Sato, Tomohiro Handa, Kiminobu Tanizawa, Toyohiro Hirai

**Affiliations:** 1 Department of Critical Care Nursing, Graduate School of Medicine, Kyoto University, 53 Shogoin Kawahara-cho, Sakyo-ku, Kyoto, 606-8507 Japan; 2 Department of Advanced Medicine for Respiratory Failure, Graduate School of Medicine, Kyoto University, 54 Shogoin Kawahara-cho, Sakyo-ku, Kyoto, 606-8507 Japan; 3 Department of Respiratory Medicine, Graduate School of Medicine, Kyoto University, 54 Shogoin Kawahara-cho, Sakyo-ku, Kyoto, 606-8507 Japan

**Keywords:** Interstitial lung disease, Information needs, Family caregivers, Oxygen therapy

## Abstract

**Background:**

The information needs of patients and their families regarding interstitial lung disease (ILD) have yet to be studied in detail, and few reports have examined the differences in information needs according to patient status. This study aimed to determine whether there are differences in information needs between outpatients with ILD and their family caregivers and whether these differences depend on long-term oxygen therapy use.

**Methods:**

Patients with fibrotic ILDs and their families who visited Kyoto University Hospital between February 2020 and March 2022 were recruited for this descriptive study. Fibrotic ILDs included idiopathic pulmonary fibrosis (IPF), other idiopathic interstitial pneumonias (IIPs) than IPF, connective tissue disease-associated ILD (CTD-ILD), and fibrotic hypersensitivity pneumonia. Data were obtained from electronic patient records and questionnaires. Descriptive data analyses were performed.

**Results:**

Sixty-five patients and their family caregivers were analyzed. Twenty-seven (41.5%) patients had IIPs (IPF 9 and other IIPs 18), 34 (52.3%) had CTD-ILD, and 4 (6.2%) had fibrotic hypersensitivity pneumonia. The most common relationship between the patient and their family was a spouse (67.7%), with 80% living together. The primary information needs among patients and their family caregivers were common up to the third rank but differed from the rest. Patients were interested in “when and where to contact health care providers” and “end-of-life care and advanced directives,” while family caregivers were interested in “diet and nutrition” and “care and support at home.” Patients with long-term oxygen therapy had higher needs for “end-of-life care and advanced directives” and “how to manage breathlessness, cough, and fatigue,” while the needs for “drugs for ILD” and “acute exacerbation of ILD” were relatively low. Family caregivers were interested in “diet and nutrition” in the long-term oxygen therapy group and “acute exacerbation of ILD” in the no long-term oxygen therapy group.

**Conclusions:**

This study found that the information needs of patients and their family caregivers were not the same and that the aspect of information needs differed by long-term oxygen therapy status. Healthcare providers should consider the position of the recipient of information, the appropriate time based on the patient’s condition, and the necessary information.

## Background

Interstitial lung disease (ILD) is a group of diffuse parenchymal lung disorders, including idiopathic pulmonary fibrosis (IPF), with a poor prognosis. The median survival time of patients with IPF is approximately 3–4 years [1, 2].

Patients with ILD and their family members often have limited information about the disease during this period [3–8]. A questionnaire-based survey reported that two-thirds of respondents (patients with pulmonary fibrosis or their caregivers) reported an apparent lack of information about their diseases at diagnosis [4]. Furthermore, a 2021 study using semi-structured qualitative interviews reported that patients with systemic sclerosis-associated ILD (SSc-ILD) and their caregivers expressed the need for clear information about SSc-ILD [5]. In contrast, several studies have reported that physicians believe they provide sufficient information to patients and caregivers [6, 9, 10].

An electronic survey conducted in 2019 found that the top information needs of patients and caregivers were consistent, while other needs differed [11]. However, the study was limited to participants with access to the Internet, the sample size of caregivers was small, and it was unclear whether the enrolled caregivers were caregivers of the patients who answered the questions. The experiences and feelings of patients and their family caregivers change throughout the disease [12–15]. For example, it has been reported that when long-term oxygen therapy (LTOT) is used due to the progression of a patient’s disease, patients often lose hope [13] and their families feel anxious, sad [14], and have an increased burden of care [15]. Therefore, the information needs of patients and their family caregivers would differ depending on the patient’s situation. However, few reports are available in this regard [8]. This descriptive study aimed to address the gap in research by examining whether there are differences in informational needs between outpatients with ILD and their family caregivers and if the patients being on LTOT affects the choices.

## Methods

### Study design and population

All patients with fibrotic ILDs who visited the ILD clinic at Kyoto University Hospital between February 2020 and March 2022 were recruited in this descriptive study when they visited the clinic with family members who were their caregivers. One family caregiver was recruited for each patient. If there were more than two family members, the family members were asked to consult with each other to determine one respondent. Fibrotic ILDs included IPF, other idiopathic interstitial pneumonias (IIPs) than IPF, connective tissue disease-associated ILD (CTD-ILD), and fibrotic hypersensitivity pneumonia (HP). ILDs were diagnosed based on guidelines for individual diseases [16–22]. Two respiratory physicians treated the patients in this study. The respiratory medicine department at the facility where the study was conducted, from where the two physicians belonged to, held a weekly conference to evaluate and review the patients. The exclusion criteria were as follows: (1) patients who had lung cancer; (2) those younger than 20 years of age; (3) patients or family caregivers who had difficulty communicating; (4) those who did not attend to the patient. The patients and caregivers provided written informed consent for participation in the study and the publication of their data. The Kyoto University Graduate School and Faculty of Medicine Ethics Committee approved the study protocol (registration number R2252).

### Measurements

Data were obtained from electronic patient records and questionnaires. Patient data, including age, sex, body mass index, anamnesis, medications, date of diagnosis of ILD, respiratory disease-related hospitalization, LTOT, and pulmonary function tests, were obtained from electronic patient records. In Japan, physicians document instructions for LTOT in the patient’s electronic medical record, allowing the verification of the presence or absence of LTOT. The composite physiology index (CPI) was used to predict the extent of fibrosis on high-resolution computed tomography. The formula used is as follows: CPI = 91.0 − (0.65 × percentage of predicted diffusing capacity of the lung for carbon monoxide) − (0.53 × percentage of predicted forced vital capacity) + (0.34 × percentage of predicted forced expiratory volume in 1 s) [23].

The questionnaire for patients was designed to collect the following information: experience as a medical worker, LTOT (during exertion or all of the time), modified Medical Research Council (mMRC) dyspnea scale, intensity and frequency of cough evaluated using a 100-mm visual analog scale (0, no cough; 100, unbearable) [24], and informational needs. Family caregivers were asked to provide the following information: age, sex, experience as a medical worker, educational status, relationship with the patient, whether they live with the patient, hours of care, and information needs. The response options for the 23 information needs in the questionnaire were derived from qualitative and survey studies [11, 13, 25–28], and the response options were validated by an ILD expert (T.H.). Patients and their family caregivers were asked to select their primary three information needs without ranking them. They did not have to respond to all three information needs if they preferred. The questionnaire survey was administered by an impartial researcher not involved in medical treatment who provided the predetermined explanatory content and distributed and collected the questionnaires. This ensured that interviewer bias was avoided. Procedure advice was obtained from the Institute for Advancement of Clinical and Translational Science at Kyoto University Hospital during the questionnaire development process. Furthermore, a pilot survey was conducted to understand the legibility of the patients and their family caregivers before drawing the final questionnaire.

### Statistical analysis

The data analyses were descriptive, and no statistical tests were performed. The data are expressed as numbers with percentages or medians with interquartile ranges. Descriptive statistics were calculated using SPSS® software version 25 (IBM Corp., Armonk, NY, USA), analyzed separately for patients and family caregivers, and compared based on whether the patients were on LTOT.

## Results

Sixty-six patients and their family caregivers were enrolled in the study; however, 1 patient and their family caregiver were excluded based on the exclusion criteria, resulting in an analysis of 65 patients and their family caregivers. Table 1 shows the characteristics of the included patients overall and in the presence or absence of LTOT. The median patient age was 74, and 38 (58.5%) were men. Twenty-seven (41.5%) patients had IIPs (IPF 9 [33.3%] and other IIPs 18 [66.7%]), 34 (52.3%) had CTD-ILD, and 4 (6.2%) had fibrotic HP. Among the ILD classifications, IIPs were more common in the group with LTOT (LTOT group, 8 [47.1%] patients), while CTD-ILD was more common in the group without LTOT (no-LTOT group, 28 [58.3%] patients). The LTOT group had a higher CPI, percentages of grades 3–4 in mMRC, and cough intensity and frequency than the no-LTOT group. Regarding the time since diagnosis, 2 (11.8%) patients in the LTOT group and 24 (50%) in the no-LTOT group had been diagnosed for less than 3 years.

**Table 1:** Characteristics of patients with interstitial lung disease by use of oxygen therapy

	Total (*n* = 65)		LTOT (*n* = 17)		no-LTOT (*n* = 48)	
Age, years, median (IQR)	74 (69–81)		72 (67–78)		74 (70–81)	
Men, n (%)	38 (58.5)		10 (58.8)		28 (58.3)	
Experience as a medical worker, n (%)	2 (3.1)		0 (0.0)		2 (4.2)	
Body mass index, median (IQR)^a^	23 (19–25)		20 (17–24)		23 (20–26)	
Classification of ILD, n (%)						
IIPs	27 (41.5)		8 (47.1)		19 (39.6)	
CTD-ILD	34 (52.3)		6 (35.3)		28 (58.3)	
CHP	4 (6.2)		3 (17.6)		1 (2.1)	
Composite physiologic index, median (IQR)^b^	45 (31–54)		62 (53–67)		42 (29–51)	
mMRC dyspnea scale, n (%)						
0	13 (20.0)		0 (0.0)		13 (27.1)	
1	6 (9.2)		1 (5.9)		5 (10.4)	
2	9 (13.8)		0 (0.0)		9 (18.8)	
3	31 (47.7)		12 (70.6)		19 (39.6)	
4	6 (9.2)		4 (23.5)		2 (4.2)	
VAS score for cough, mm, median (IQR)						
Intensity	31 (13–60)		59 (26–67)		28 (6–50)	
Frequency	25 (5–50)		40 (13–76)		22 (3–49)	
Duration of disease, year, n (%)						
< 1	11 (16.9)		0 (0.0)		11 (22.9)	
1≤, < 2	7 (10.8)		1 (5.9)		6 (12.5)	
2≤, < 3	8 (12.3)		1 (5.9)		7 (14.6)	
3≤, < 4	3 (4.6)		2 (11.8)		1 (2.1)	
4≤, < 5	5 (7.7)		2 (11.8)		3 (6.3)	
≥ 5	31 (47.7)		11 (64.7)		20 (41.7)	
Respiratory disease-related hospitalization						
History, n (%)	46 (70.8)		15 (88.2)		31 (64.6)	
Number, median (IQR)^c^	1 (0–2)		2 (1–4)		1 (0–2)	
Acute exacerbation, n (%)^c^	8 (12.5)		2 (12.5)		6 (12.5)	
Antifibrotic agents, n (%)	12 (18.5)		7 (41.2)		5 (10.4)	
Oxygen, n (%)						
During exertion			2 (11.8)			
All of the time			15 (88.2)			

*CHP* chronic hypersensitivity pneumonia, *CTD-ILD* connective tissue disease-associated interstitial lung disease, *IIPs* idiopathic interstitial pneumonias, *ILD* interstitial lung disease, *IQR* interquartile range, *mMRC* modified Medical Research Council, *LTOT* long-term oxygen therapy, *VAS* visual analog scale. ^a^Total (*n* = 59); LTOT (*n* = 17); no-LTOT (*n* = 42), ^b^Total (*n* = 43); LTOT (*n* = 7); no-LTOT (*n* = 36), ^c^Total (*n* = 64); LTOT (*n* = 16); no-LTOT (*n* = 48)

Table 2 shows the family caregivers’ characteristics and association with LTOT status. Their median age was 70, and 12 (18.5%) family caregivers were men. The most common relationship with the patient was a spouse (44 [67.7%]), with 52 (80%) family caregivers living with their patients. The median age of family caregivers of patients in the LTOT and no-LTOT groups was 68 and 72, respectively. The percentage of family caregivers living with their patients was 13 (76.5%) in the LTOT group and 31 (64.6%) in the no-LTOT group.

**Table 2:** Family caregivers’ characteristics of patients with interstitial lung disease by use of oxygen therapy

	Total (*n* = 65)		LTOT (*n* = 17)		no-LTOT (*n* = 48)	
Age, years, median (IQR)	70 (57–76)		68 (53–75)		72 (61–76)	
Men, n (%)	12 (18.5)		3 (17.6)		9 (18.8)	
Experience as a medical worker, n (%)^a^	9 (13.8)		1 (5.9)		8 (17.0)	
Educational status, n (%)						
Junior high school	9 (13.8)		2 (11.8)		7 (14.6)	
High school	26 (40.0)		7 (41.2)		19 (39.6)	
Junior college or vocational school	17 (26.2)		3 (17.6)		14 (29.2)	
College	13 (20.0)		5 (29.4)		8 (16.7)	
Graduate School	0 (0.0)		0 (0.0)		0 (0.0)	
Relationship, n (%)						
Parent	3 (4.6)		0 (0.0)		3 (6.3)	
Spouse	44 (67.7)		11 (64.7)		33 (68.8)	
Child	14 (21.5)		4 (23.5)		10 (20.8)	
Relative	3 (4.6)		2 (11.8)		1 (2.1)	
Friend	0 (0.0)		0 (0.0)		0 (0.0)	
Others	1 (1.5)		0 (0.0)		1 (2.1)	
living together, n (%)	52 (80.0)		13 (76.5)		31 (64.6)	
Hours of care, median (IQR)	0 (0–2)		1 (0–3)		0 (0–2)	

*IQR* interquartile range, *LTOT* long-term oxygen therapy. ^a^Total (*n* = 64); LTOT (*n* = 17); no-LTOT (*n* = 47)

**Fig. 1:**
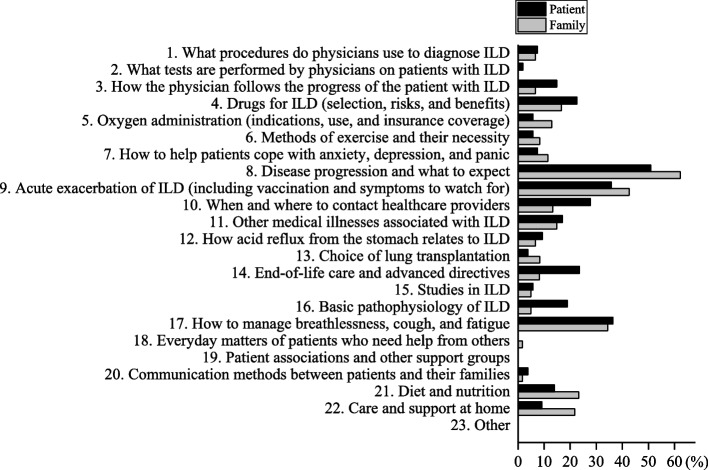
Information needs of patients with interstitial lung disease and their families. The top five information needs of patients with interstitial lung disease were “disease progression and what to expect,” “how to manage breathlessness, cough, and fatigue,” “acute exacerbation of ILD,” “when and where to contact healthcare providers,” and “end-of-life care and advance directives.” The top five information needs of the family were “Disease progression and what to expect,” “Acute exacerbation of ILD,” “How to manage breathlessness, cough, and fatigue,” “Diet and nutrition,” and “Care and support at home.” ILD, interstitial lung disease. Information need number (INF) 1, 2, 4, 6, 12, 13, 15, 16, 18, 19, 20, 23 (Patient, *n* = 53; Family, *n* = 60), INF 7, 8, 11 (Patient, *n* = 53; Family, *n* = 61), INF 5 (Patient, *n* = 53; Family, *n* = 62), INF 3, 10 (Patient, *n* = 54; Family, *n* = 60), INF 22 (Patient, *n* = 55; Family, *n* = 60), INF 14, 17 (Patient, *n* = 55; Family, *n* = 61), INF 9 (Patient, *n* = 56; Family, *n* = 61), INF 21 (Patient, *n* = 57; Family, *n* = 60)

As shown in Fig. 1, the principal information needs of patients were “disease progression and what to expect,” “how to manage breathlessness, cough, and fatigue,” “acute exacerbation of ILD,” “when and where to contact health care providers,” and “end-of-life care and advanced directives,” in that order. In contrast, the primary information needs of family caregivers were “disease progression and what to expect,” “acute exacerbation of ILD,” “how to manage breathlessness, cough, and fatigue,” “diet and nutrition,” and “care and support at home,” in that order. Of the top five participant-selected items, we focused on those that differed between the patients and family caregivers. “When and where to contact health care providers” (15 [27.8%] in the patient group vs. 8 [13.3%] in the family caregiver group) and “end-of-life care and advanced directives” (13 [23.6%] in the patient group vs. 5 [8.2%] in the family caregiver group) were of high interest to patients and low interest to family caregivers. In contrast, “diet and nutrition” (8 [14.0%] in the patient group vs. 14 [23.3%] in the family caregiver group) and “care and support at home” (5 [9.1%] in the patient group vs. 13 [21.7%] in the family caregiver group) was of high interest to the family caregivers and low interest to the patients. “Disease progression and what to expect” was the top information need among patients and family members, although there was a difference of more than 10%.

**Fig. 2:**
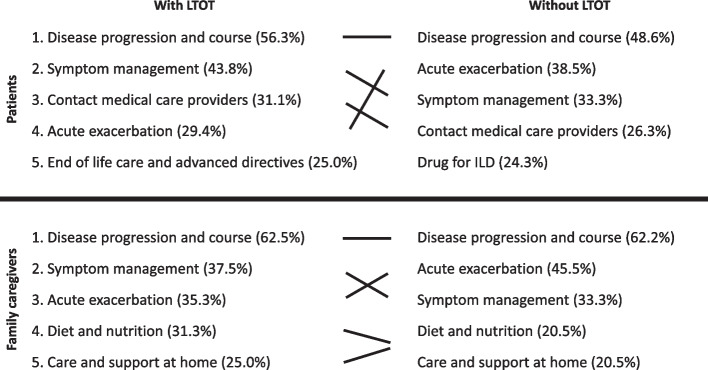
Principal information needs of patients with interstitial lung disease and their caregivers by oxygen therapy. Patients and their family caregivers were concerned with disease progression and course, with or without LTOT. Patients in the LTOT group had higher needs for symptom management, contact medical care providers, and end-of-life care and advanced directives, while the needs for acute exacerbation and drugs were relatively low. Family caregivers were interested in diet and nutrition and care and support at home, and acute exacerbation in the no-LTOT group. ILD, interstitial lung disease; LTOT, long-term oxygen therapy

Tables 3 and 4 show the information needs of patients and family caregivers with and without LTOT, respectively. The principal information needs of patients with and without LTOT differ in the following ways: (1) “drugs for ILD” ranked fifth in the no-LTOT group and outside fifth place in the LTOT group; (2) “acute exacerbation of ILD” ranked fourth in the LTOT group and second in the no-LTOT group; and (3) “end-of-life care and advanced directives” ranked fifth in the LTOT group and outside fifth place in the no-LTOT group. Of the five principal items, those that differed were noted: for patients, “acute exacerbation of ILD” (5 [29.4%] in the LTOT group vs. 15 [38.5%] in the no-LTOT group) and “how to manage breathlessness, cough, and fatigue” (7 [43.8%] in the LTOT group vs. 13 [33.3%] in the no-LTOT group), and for family members, “acute exacerbation of ILD” (6 [35.3%] in the LTOT group vs. 20 [45.5%] in the no-LTOT group) and “diet and nutrition” (5 [31.3%] in the LTOT group vs. 9 [20.5%] in the no-LTOT group). The principal information needs of patients with interstitial lung disease and their family caregivers by use of LTOT are shown in Fig. 2.

**Table 3:** Information needs of patients with interstitial lung disease by use of oxygen therapy

	LTOT (*n* = 17)			no-LTOT (*n*= 48)		
	Rank	n	%	Rank	n	%
What procedures do physicians use to diagnose ILD^a^		1	6.3		3	8.1
What tests are performed by physicians on patients with ILD^a^		0	0.0		1	2.7
How the physician follows the progress of the patient with ILD^b^		3	18.8		5	13.2
Drugs for ILD (selection, risks, and benefits)^a^		3	18.8	5	9	24.3
Oxygen administration (indications, use, and insurance coverage)^a^		1	6.3		2	5.4
Methods of exercise and their necessity^a^		0	0.0		3	8.1
How to help patients cope with anxiety, depression, and panic^a^		2	12.5		2	5.4
Disease progression and what to expect^a^	1	9	56.3	1	18	48.6
Acute exacerbation of ILD (including vaccination and symptoms to watch for)^d^	4	5	29.4	2	15	38.5
When and where to contact healthcare providers^b^	3	5	31.3	4	10	26.3
Other medical illnesses associated with ILD^a^		2	12.5		7	18.9
How acid reflux from the stomach relates to ILD^a^		1	6.3		4	10.8
Choice of lung transplantation^a^		1	6.3		1	2.7
End-of-life care and advanced directives^c^	5	4	25.0		9	23.1
Studies in ILD^a^		1	6.3		2	5.4
Basic pathophysiology of ILD^a^		2	12.5		8	21.6
How to manage breathlessness, cough, and fatigue^c^	2	7	43.8	3	13	33.3
Everyday matters of patients who need help from others^a^		0	0.0		0	0.0
Patient associations and other support groups^a^		0	0.0		0	0.0
Communication methods between patients and their families^a^		1	6.3		1	2.7
Diet and nutrition^e^		0	0.0		8	19.5
Care and support at home^c^		1	6.3		4	10.3
Other^a^		0	0.0		0	0.0

*ILD* interstitial lung disease, *LTOT* long-term oxygen therapy.^a^LTOT (*n* = 16); no-LTOT (*n* = 37); ^b^LTOT (*n* = 16); no-LTOT (*n* = 38); ^c^LTOT (*n* = 16); no-LTOT (*n* = 39); ^d^LTOT (*n* = 17); no-LTOT (*n* = 39); ^e^LTOT (*n* = 16); no-LTOT (*n* = 41)

**Table 4:** Information needs of families of patients with interstitial lung disease by use of oxygen therapy

	LTOT (*n* = 17)			no-LTOT (*n* = 48)		
	Rank	n	%	Rank	n	%
What procedures do physicians use to diagnose ILD^a^		1	6.3		3	6.8
What tests are performed by physicians on patients with ILD^a^		0	0.0		0	0.0
How the physician follows the progress of the patient with ILD^a^		1	6.3		3	6.8
Drugs for ILD (selection, risks, and benefits)^a^		2	12.5		8	18.2
Oxygen administration (indications, use, and insurance coverage)^d^		2	12.5		6	13.0
Methods of exercise and their necessity^a^		1	6.3		4	9.1
How to help patients cope with anxiety, depression, and panic^b^		1	6.3		6	13.3
Disease progression and what to expect^b^	1	10	62.5	1	28	62.2
Acute exacerbation of ILD (including vaccination and symptoms to watch for)^c^	3	6	35.3	2	20	45.5
When and where to contact healthcare providers^a^		1	6.3		7	15.9
Other medical illnesses associated with ILD^c^		2	11.8		7	15.9
How acid reflux from the stomach relates to ILD^a^		0	0.0		4	9.1
Choice of lung transplantation^a^		3	18.8		2	4.5
End-of-life care and advanced directives^b^		2	12.5		3	6.7
Studies in ILD^a^		2	12.5		1	2.3
Basic pathophysiology of ILD^a^		1	6.3		2	4.5
How to manage breathlessness, cough, and fatigue^b^	2	6	37.5	3	15	33.3
Everyday matters of patients who need help from others^a^		0	0.0		1	2.3
Patient associations and other support groups^a^		0	0.0		0	0.0
Communication methods between patients and their families^a^		0	0.0		1	2.3
Diet and nutrition^a^	4	5	31.3	4	9	20.5
Care and support at home^a^	5	4	25.0	4	9	20.5
Other^a^		0	0.0		0	0.0

*ILD* interstitial lung disease, *LTOT* long-term oxygen therapy.^a^LTOT (*n* = 16); no-LTOT (*n* = 44); ^b^LTOT (*n* = 16); no-LTOT (*n* = 45); ^c^LTOT (*n* = 17); no-LTOT (*n* = 44); ^d^LTOT (*n* = 16); no-LTOT (*n* = 46)

## Discussion

Our study is the first to investigate differences in information needs between patients with ILD and their family caregivers, depending on whether the patient was using LTOT. Patients and caregivers shared interests in “disease progression and what to expect,” “how to manage breathlessness, cough, and fatigue,” and “acute exacerbation of ILD,” but differed from the rest. Among the different information needs, patients were interested in “when and where to contact health care providers” and “end-of-life care and advanced directives,” while family caregivers were interested in “diet and nutrition” and “care and support at home.” Patients in the LTOT group had higher needs for “end-of-life care and advanced directives” and “how to manage breathlessness, cough, and fatigue,” while the needs for “drugs for ILD” and “acute exacerbation of ILD” were relatively low. Family caregivers were interested in “diet and nutrition” in the LTOT group, while “acute exacerbation of ILD” in the no-LTOT group.

The most important piece of information needed by patients and their families was consistent with the results of an Internet survey of patients with IPF and caregivers [11], but the need for other information varied. We believe that the reason the first need for “disease progression and what to expect” was unanimous is due to the diversity of the disease course in ILD, reflecting both groups’ uncertainties regarding the future of the patient’s condition [16]. We suggest two reasons why the principal needs, excluding the most important, differed across the studies. First, the target population differed; our study targeted patients with ILD, whereas the Internet survey targeted patients with IPF. Second, some of the information needs options differed between the studies. Our study used the information needs options in the Internet survey and additional seven options based on those included in other studies to identify potential information needs [13, 25–28].

Among the five information needs selected as the most important among patients and family caregivers, we focused on those with the largest discrepancy. Patients were interested in “when and where to contact health care providers” and “end-of-life care and advance directives.” Based on the results of this study on mMRC, 80% of patients had subjective symptoms, which may indicate that the patients were aware of the disease. It is also possible that patients are aware of the poor prognosis of ILD [1]. Therefore, patients may be interested in these two information needs based on their own knowledge and experience of the disease. In contrast, family caregivers were interested in “diet and nutrition” and “care and support at home,” with the LTOT group being particularly interested in “diet and nutrition” more than the no-LTOT group. Given the large proportion of spouses (67.7%) and living together (80.0%) in the family-patient relationship and that approximately 80% of the LTOT group lived together, we assumed that these options were chosen because the family respondents in this study are the primary cooks and caregivers in the home. Furthermore, dietary issues have been reported to be a challenge for caregivers when caring for patients at home [29]. Nutrition awareness has increased recently, with studies showing an association between weight loss and ILD prognosis [30, 31]. However, the lower importance rating by the patients regarding nutrition in this study suggests that physicians may not adequately explain nutrition to their patients, resulting in a lack of awareness.

For patients and family caregivers, “acute exacerbation of ILD” was selected more often in the no-LTOT group than in the LTOT group, ranking second. This may be related because a larger proportion of patients in the no-LTOT group had been diagnosed with ILD for less than 1 year. Acute exacerbations were less frequently perceived by patients as explained by the physician at diagnosis; however, physicians believed that they explained it to the patients [10]. Thus, with a shorter time since diagnosis, the no-LTOT group may have a reduced perception of being briefed about acute exacerbations, perhaps leading to the information requirement being selected more often. In addition, patients in the non-LTOT group had less severe disease, as shown in Table 2; thus, they may have been less bothered by symptoms than those in the LTOT group. Consequently, the potential risk of acute exacerbation may be relatively focused on in the non-LTOT group.

The reason “drugs for ILD” was not in the top five in the LTOT group but was in the top five in the no-LTOT group may be due to the higher percentage of patients in the LTOT group (41.2%) already using antifibrotic medications. In addition, the higher percentage of patients with ILD in the no-LTOT group who had been diagnosed less than 3 years, as well as the higher percentage of patients with milder symptoms based on CPI, mMRC, and visual analog scale (cough intensity and frequency), may explain the higher interest in future drug treatments. Patients value early commencement of drug treatment [10], and some reports have demonstrated the efficacy of antifibrotic agents in mild and moderate cases [32] and in progressive fibrosing interstitial lung disease [33]. It was also reported that antifibrotic agents can be used safely in patients being treated with anticoagulants [34]. Considering these factors, we believe drug treatment should be explained to patients as early as possible after diagnosis and initiated based on a mutual understanding among healthcare providers, patients, and their family caregivers.

The fact that “end-of-life care and advanced directives” ranked fifth in the LTOT group, which was outside the top five in the no-LTOT group, may indicate more severe disease symptoms in the LTOT group, causing patients to be more interested in end-of-life care. A study of patients with progressive idiopathic fibrotic ILD reported that patients and caregivers recognized the importance of end-of-life care planning conversations but did not know how to initiate the conversation, highlighting a sense of unease regarding the topic [26]. However, the percentage of patients in the no-LTOT group who chose “end-of-life care and advance directives” was 23.1%, almost unchanged from the 25.0% in the LTOT group. The slightly lower value in the no-LTOT group may be because the no-LTOT group’s interest was spread over other information. In addition, considering the uncertain trajectory of ILDs, questions regarding early intervention in end-of-life care plans are warranted [35]. Therefore, considering the presence or absence of LTOT, asking about individual preferences, and providing early information about end-of-life care and advanced directives are necessary.

Moreover, the LTOT patient group was more interested in information such as “how to manage breathlessness, cough, and fatigue” than the no-LTOT group. This information needs may be higher in the LTOT group because the patients had more subjective symptoms and greater severity of illness. A qualitative study for a population that included 90% of patients using LTOT also reported that many patients indicated a need for practical information to help them manage their illness [13]. Therefore, more information on managing breathlessness, cough, and fatigue should be provided to patients using LTOT to increase their confidence and ability to self-manage.

This study had some limitations. First, subgroup analyses according to disease category could not be performed because the number of patients in each disease category was low. Second, this study was conducted during the coronavirus disease 2019 pandemic; thus, the study sites prohibited family members from entering the treatment rooms during the study period. Consequently, the sample size was small, which makes generalizing our results difficult. Additionally, the study was conducted at a single institution in Japan, which may also limit the generalizability of our results. Third, clinical sensitivity testing was not conducted to assess the questionnaire’s comprehensiveness, clarity, and face validity [36]. However, the questionnaire was reviewed by an ILD specialist and pilot-tested with eligible participants during the questionnaire development phase in this study. Fourth, regarding information, patients were not asked how they obtained information or by what means they preferred to obtain it. Patients often use internet search engines to obtain disease-related information [9], which is reported to be inadequate, inaccurate, and undated [37, 38]. Future multicenter studies with larger sample sizes and more questions regarding information sources are needed, with testing to improve the validity of the questionnaire. Finally, a descriptive study with a cross-sectional approach could not assess the sequential changes in the individual patients as their disease progressed. A further prospective and longitudinal study in the mild ILD patient group may be required to understand the patient journey of ILD more precisely from the viewpoints of both patients and caregivers.

## Conclusions

Information needs differed between patients with ILD and their family caregivers and depended on LTOT status. Healthcare providers should consider the recipient’s position (patients or caregivers), the appropriate time based on the patient’s condition, and the necessary information when communicating information. This would ensure that all patients and their caregivers receive the required information, reducing their concerns and improving their quality of life. Future research should investigate which delivery methods patients and their family caregivers prefer and are most effective and include longitudinal studies based on changes in the patient’s conditions.

## Data Availability

The datasets generated and/or analyzed during the current study are not publicly available because of limitations on secondary use but are available from the corresponding author upon reasonable request.

## Abbreviations

CPI: composite physiological index
CTD-ILD: connective tissue disease-associated interstitial lung disease
HP: hypersensitivity pneumonia
IIPs: idiopathic interstitial pneumonias
ILD: interstitial lung disease
IPF: idiopathic pulmonary fibrosis
mMRC: modified Medical Research Council
LTOT: long-term oxygen therapy
SSc: systemic sclerosis
